# Schiff Bases of Indoline-2,3-dione: Potential Novel Inhibitors of Mycobacterium Tuberculosis (Mtb) DNA Gyrase ^†^

**DOI:** 10.3390/molecules16097864

**Published:** 2011-09-13

**Authors:** Tarek Aboul-Fadl, Hatem A. Abdel-Aziz, Mohammed K. Abdel-Hamid, Tilal Elsaman, Jane Thanassi, Michael J. Pucci

**Affiliations:** 1 Department of Pharmaceutical Chemistry, College of Pharmacy, King Saud University, P.O. Box 2457, Riyadh 11451, Saudi Arabia; 2 Department of Pharmaceutical Medicinal Chemistry, Faculty of Pharmacy, Assiut University, Assiut 71526, Egypt; 3 Antimicrobial Drug Discovery, Achillion Pharmaceuticals 300 George Street New Haven, CT 06511, USA

**Keywords:** Schiff bases, Indoline-2,3-dione, microwave irradiation, *Mycobacterium tuberculosis *(Mtb), Mtb DNA gyrase, MOE

## Abstract

In the present study a series of Schiff bases of indoline-2,3-dione were synthesized and investigated for their Mtb gyrase inhibitory activity. Promising inhibitory activity was demonstrated with some of these derivatives, which exhibited IC_50_ values ranging from 50–157 μM. The orientation and the ligand-receptor interactions of such molecules within the Mtb DNA gyrase A subunit active site were investigated applying a multi-step docking protocol using Molecular Operating Environment (MOE) and Autodock4 docking software. The results revealed the importance of the isatin moiety and the connecting side chain for strong interactions with the enzyme active site. Among the tested compounds the terminal aromatic ring benzofuran showed the best activity. Promising new leads for developing a novel class of Mtb gyrase inhibitors were obtained from Schiff bases of indoline-2,3-dione.

## 1. Introduction

Infections caused by mycobacteria are the single largest cause of death worldwide. Fluoroquinolones have been used with limited success as part of a second-line chemotherapeutic regime against mycobacterial diseases. With the global emergence of multidrug-resistant tuberculosis (MDRTB) and extensively drug-resistant tuberculosis (XDRTB) there is an urgent need to develop new anti-mycobacterials. Design of new inhibitors with greater efficacy against mycobacterial enzymes would facilitate the development of efficient anti-TB drugs [1]. *Mycobacterium tuberculosis* (Mtb), the aetiologic agent of tuberculosis, is unusual in that it possesses only one type II topoisomerase, DNA gyrase [2]. Consequently, the Mtb DNA gyrase exhibits a different activity spectrum as compared to other DNA gyrases. Despite the fact that it supercoils DNA with an efficiency comparable to that of other DNA gyrases it shows enhanced relaxation, DNA cleavage, and decatenation activities [3].

DNA gyrase consists of two subunits, gyrase A (GyrA) and gyrase B (GyrB), that together form a functional heterodimer structure A2B2. While the function of the GyrA subunit is primarily the breakage and reunion of the bacterial DNA, the GyrB subunit possesses an ATP-ase activity. In the absence of the ATP, DNA gyrase catalyzes only the relaxation of supercoiled DNA but not the introduction of negative supercoils [4,5].

Quinolones are the only class of the DNA gyrase inhibitors currently used in clinical practice. They act by inhibiting the GyrA subunit, thus interfering with the DNA cleavage and ligation reactions [6]. The coumarins (e.g., novobiocin) and cyclothialidines (e.g., GR122222X), natural antibiotics from the *Streptomyces* organisms, are the most studied inhibitors of the GyrB subunit [7]. Both classes act as competitive inhibitors of the ATP-binding site on the GyrB subunit, thus inhibiting the ATP-dependent step in the enzyme catalytic cycle [8]. The inhibitory mechanism has also been characterized with radiolabelled benzoylcyclothialidine and dihydronovobiocin and by structural analysis [9]. More recently, GyrB inhibitors from various chemical classes have been reported including indazoles [10], pyrazoles [11], benzimidazoles [12], phenols [13] and indolinones [14]. Furthermore, gyrase inhibitors prepared from indoline-2,3-dione (isatin) derivatives have been also reported [15,16], in addition to its Schiff bases, displaying anti-TB activity [17]. Accordingly isatin was identified as a versatile lead molecule for further design of potential gyrase inhibitors and the current work is directed to synthesize and investigate such activity based on this interesting moiety focusing on interactions with the DNA gyrase A subunit.

## 2. Results and Discussion

### 2.1. Chemistry

**Scheme 1:**
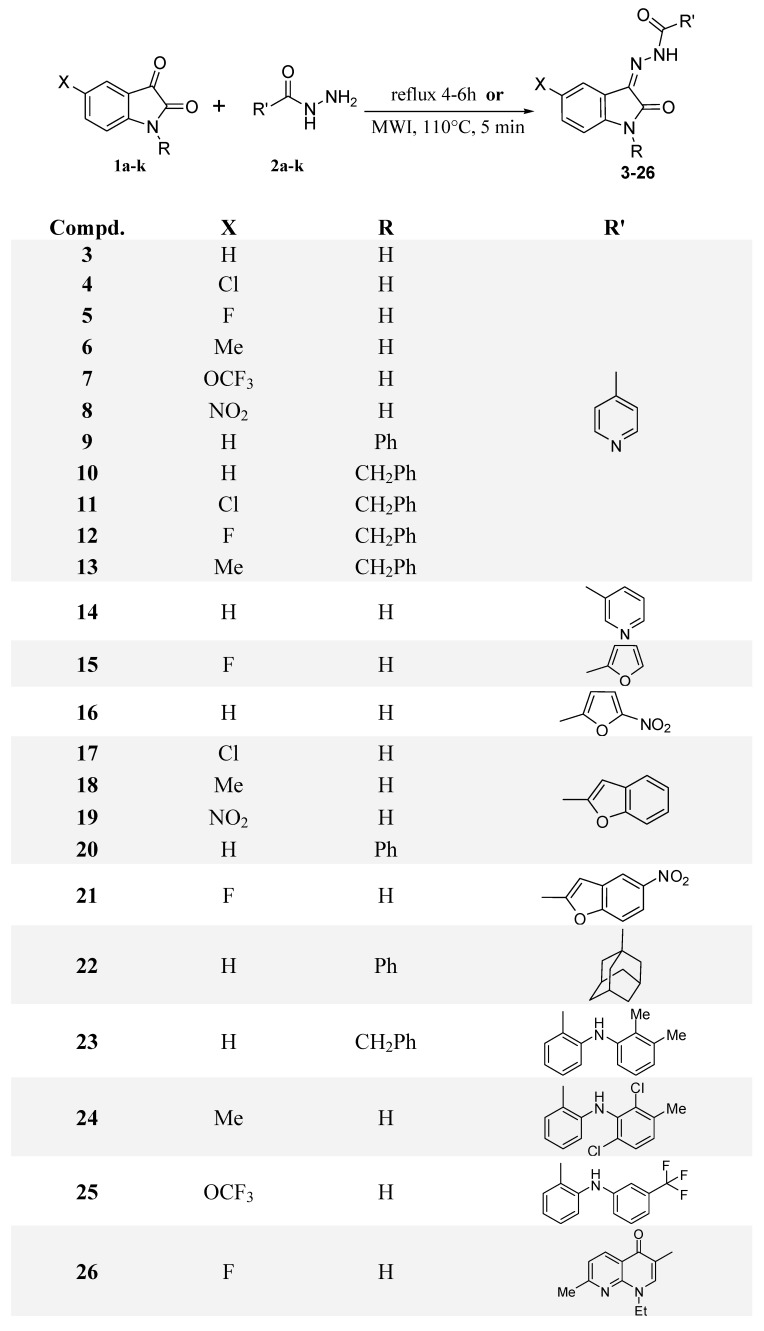
Synthesis of the targeted Schiff Bases.

The targeted hydrazones **3**-**26** were obtained from the condensation of the appropriate isatin **1a**-**k** with hydrazide derivatives **2a**-**k** in acidified ethanol for 4 to 6 hours or by using microwave irradiation (MWI) at 110 °C for 5 minutes (Scheme 1). On the other hand, the hydrazide building blocks of compounds **15-26** were prepared according the reported procedures, either conventionally or by microwave assisted synthesis [18,19]. Spectral data of the targeted compounds were consistent with their assigned structures shown in Scheme 1.

### 2.2. Mtb DNA Gyrase Inhibitory Activity

**Figure 1:**
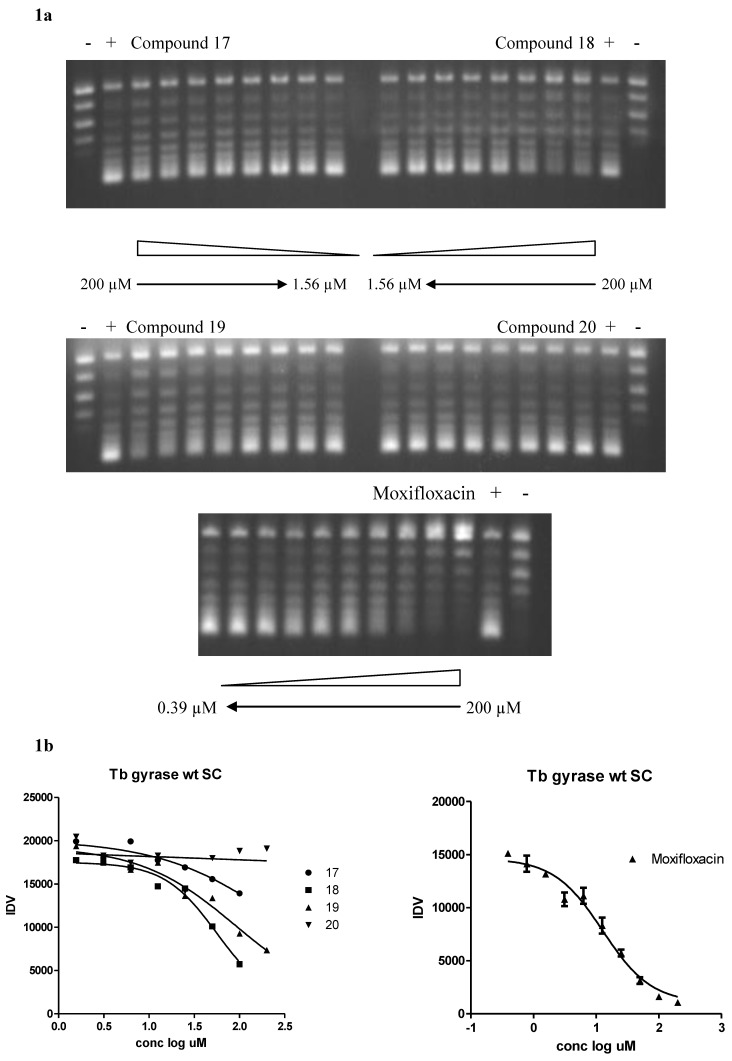
(**a**) Mtb DNA gyrase supercoiling inhibition by inhibitors and moxifloxacin. Two-fold increases in inhibitor concentrations ranging from 1.56 to 200 µM were added to pBR322 relaxed DNA in the presence of Mtb DNA gyrase enzyme. Control lanes are: Lane 1, DNA with no enzyme or inhibitor and Lane 2, DNA with enzyme but no inhibitor. IC_50_ values were calculated by measurement of fluorescence intensity after ethidium bromide staining of the supercoiling band at the bottom of the lanes. Moxifloxacin was used as an enzyme inhibitor positive control. (**b**) IC_50_ curves of supercoiling (SC) fluorescence data. DNA fluorescence was quantitated with an Alpha Imager 2200 Analysis System (Cell Biosciences, Santa Clara, CA, USA) and IC_50_ values were determined by nonlinear regression analysis with Graphpad Prism software (Graphpad Software, Inc., San Diego, CA, USA). IDV = Integrated Density Value.

The isatin Schiff bases synthesized and characterized in this report were tested for their ability to inhibit supercoiling activity of DNA gyrase. DNA gyrase is the only enzyme known so far having the ability to introduce negative supercoils in DNA. In all the species of mycobacteria, including Mtb, DNA gyrase is the sole type II topoisomerase carrying out the reactions of both gyrase and topoisomerase IV. Since the DNA gyrase from Mtb and *M. smegmatis* are highly similar at a functional as well as a protein sequence level and antigenic properties [20], Mtb strain H37RV DNA gyrase has been used for carrying out the supercoiling assays. The results of gyrase inhibition with various compounds are presented in Figure 1a and b. The concentration at which enzyme was 50% inhibited was designated as the IC_50_. Table 1 shows the IC_50_ values of the tested compounds which were found to be much higher compared to the positive control moxifloxacin. Three compounds, namely **8**, **18** and **19**, showed better inhibitory activity relative to the other tested compounds. There were no obvious structure-activity relationships among these three compounds. In addition, the effect of different substituent variations on the isatin moiety on activity was not clear. Maximum activity was shown with compound **18**, therefore, we propose that it can be utilized as a lead structure for future chemical optimization studies for novel inhibitors of the Mtb enzyme DNA gyrase.

**Table 1:** Lipophilicity (Clog P) and Mtb wt gyrase inhibitory activity of the synthesized Schiff bases of isatins.

Compd. No.	Clog P ^a^	Mtb wt gyrase IC_50_ (μM) ^b^
**3**	0.72	>200
**4**	1.4	>200
**5**	0.87	>200
**6**	1.22	>200
**7**	1.75	>200
**8**	0.47	157.3
**9**	2.64	>200
**10**	3.37	>200
**11**	4.08	>200
**12**	3.51	>200
**13**	3.87	>200
**14**	0.72	>200
**15**	1.54	>200
**16**	1.14	>200
**17**	3.49	>200 (294)
**18**	3.28	~50, 56.3
**19**	2.52	89.9
**20**	4.7	>200
**21**	2.67	>200
**22**	5.36	>200
**23**	7.29	>200
**24**	6.53	>200
**25**	6.14	>200
**26**	0.27	>200

^a^ Calculated using PC-software package (MacLogP 2.0, BioByte Corp., CA, USA);

^b^ IC_50_ of the reference drug moxifloxacin is 12.76, 11.23, 12.7 μM.

### 2.3. Molecular Modeling Study

Investigation of the orientation and the ligand-receptor interactions of such molecules within the Mtb DNA gyrase active site could provide valuable information to assist in the design of future inhibitors. This modeling study aims to guide the optimization of the tested compounds toward more potent inhibitors. With only limited literature data available on the possible active site of Mtb DNA gyrase, a multi-step docking protocol has been developed using the docking softwares Molecular Operating Environment (MOE) [21] and Autodock4 [22]. While it is not clear from the biological data if the tested derivatives inhibit either the GyrA or GyrB subunits, we choose to investigate the GyrA binding possibility. In this protocol, the recently revealed crystal structure of Mtb DNA gyrase GyrA N-terminal domain (3ilw) [23] was employed.

The first step was to use the powerful “active site finder” function of the MOE software to nominate potential docking pockets and this resulted in the identification of 16 potential pockets within the manipulated 3ilw crystal structure. These pocket sites were refined using preliminary global docking of compound **18** where the pocket that shows the highest clustering of docked poses was chosen as the docking pocket for further studies. This pocket was found to be made of 119 amino acid residues starting at Arg39 and ending at Tyr276. It is worth noting that this site is distinct but in close proximity to the pocket recently identified as the active site for fluoroquinolone derivatives [24].

The assigned pocket was used in a more sophisticated docking study using Autodock4 software. Here the graphical interface of AutoDockTools (ADT 1.5.4) software was used to optimize the enzyme and the ligands. A grid box size of 50 × 50 × 50 points was built and the box was centered on the pre-assigned pocket with a grid spacing of 0.375 Å. Four ligands, compounds **8**, **17-19**, were selected for their promising activity and docked into Mtb DNA gyrase.

The docking results revealed an average binding energy for the docked compounds in the range of −8.09 to −3.21 kcal/mol (Table 2). The calculated binding energy trend found to be in rough agreement with the biological activity. A closer look was obtained by processing the docked poses by MOE software in order to study the enzyme-ligand interactions in more details.

**Table 2:** Output for the docking of compounds **8**, **17-19**.

Compd. No.	No. of poses in major cluster	Binding energy (kcal/mol)	Hydrogen bond residues	Other contacts
8	41	−7.02	Arg98, Asn172, Ser178	Lys49
17	35	−3.21	Lys49, Ser178	-
18	63	−8.09	Lys49, Ser178, Gly179	His52, Arg98
19	48	−6.85	Lys49, Ser178, Gly179	Lys49

It was found that derivatives carrying a benzofuran moiety (compounds **17-19**) showed a similar orientation within the enzyme active site. On the other hand, derivative **8**, which carries a pyridine terminal moiety, was found to have a flip orientation inside the active site. However, this flip orientation does not prevent its interaction at the active site in the same pattern observed by derivatives **17-19** (Table 2).

**Figure 2:**
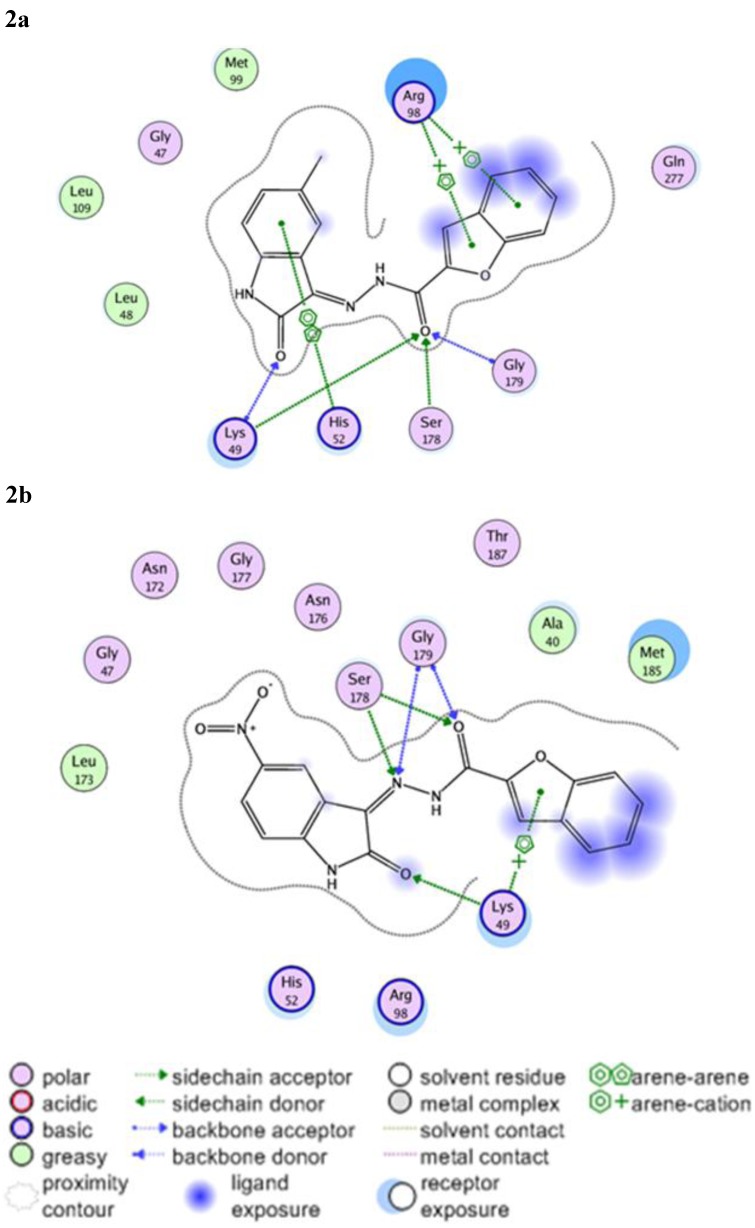
2D Representation of **18** (2a) and **19** (2b) within the 3ilw active site.

It can be noted from Table 2 that residues Lys49, Ser178 and Gly179 showed the most significant hydrogen bond interactions with the docked ligands. In case of **18** it was found that Lys49 residue, which is known to show significant interactions with fluoroquinolone-type inhibitors [24], participates in two hydrogen bond interactions with both the isatin and the exo-cyclic carbonyl groups (Figure 2a). This type of interaction can be observed for derivatives **17** and **19** as well, but with weaker bonds. In case of **8** this interaction cannot be observed, however, it is replaced by strong hydrogen bonding with Ser178. Another interaction that affecting the isatin nucleus that was observed with **18** involves a strong hydrophobic interaction with His52, a contact that was not noted with other derivatives and may provide an insight about the hydrophobic requirements for this side of the pocket. Similarly, derivative **18** shows arene-cation interactions with the positively charged Arg98 residue. In the case of **19**, because of the displacement caused by the bulky nitro-group, this interaction was lost and replaced by a weak arene-cation interaction with Lys49 (Figure 2b).

**Figure 3:**
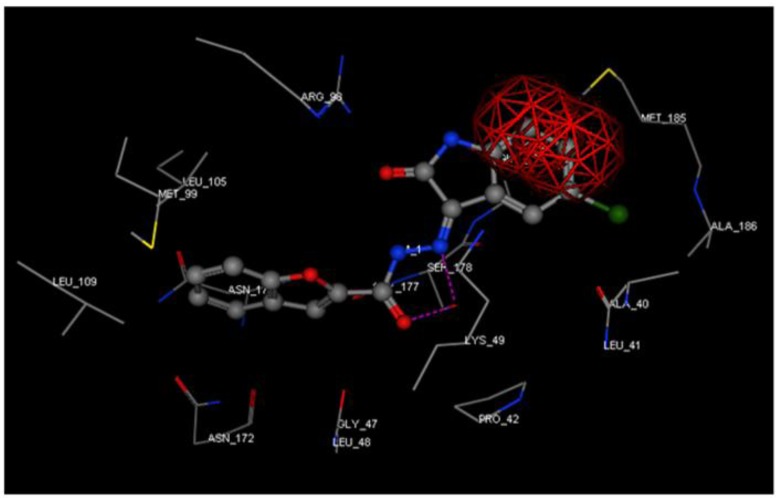
Compound **17** (ball and stick) docked into 3ilw active site. Steric clash shown as a red framework.

Such interactions cannot be observed for derivative **17**, despite the structural similarity with **18** and **19**. For reasons that remain unclear, **17 **failed to orient into the active pocket showing a steric clash between the isatin benzo moiety and Met185 residue (Figure 3).

The modeling results showed the importance of the isatin moiety and the connecting side chain for strong interactions with the enzyme active site. However, substituents carried by the isatin ring seem to also play a crucial role for activity. While only substituents at position 5 of the isatin ring were attempted, substitution at other positions will be investigated in future studies. In addition, the benzofuran provided the best activity as the terminal aromatic ring, but we believe that hydrophobic substituents on this ring may have a positive effect on the inhibitory activity.

Computation of the log P was based on the fragment method developed by Leo contained in a PC-software package [25]. It was found that there is no obvious relation between the gyrase inhibitory activity of the tested compounds and their lipophilicity. Clearly the lipophilicity has an influence on the activity, but it does not solely determine the gyrase activity of these compounds.

## 3. Conclusions

This work describes the synthesis and investigation of Mtb gyrase inhibitory activity of some Schiff bases of indoline-2,3-dione. Preliminary biological evaluation demonstrated that some of these compounds possess notable Mtb gyrase inhibitory activity, suggesting potential for the development of new class of Mtb gyrase inhibitors. In addition, a pharmacophore based study was performed to explain the biological activity on structural bases. The modeling results showed the importance of the isatin moiety and the connecting side chain for strong interactions with the enzyme active site. However, substituents carried by the isatin ring seem to play a crucial role for activity. While only substituents at position 5 of the isatin ring were attempted, substitution at other positions will be investigated in future studies. Furthermore, as terminal aromatic ring the benzofuran moiety provided the best activity, but we believe that hydrophobic substituents on this ring may have a positive effect on the inhibitory activity. On the bases of the obtained results, current research is in progress to improve the Mtb gyrase inhibitory activity/selectivity profile of this chemotype. In addition, further research will investigate whether these inhibitors are GyrA or GyrB using *in vitro* methods.

## 4. Experimental

### 4.1. General

Isatin building blocks **1**(**a**-**f**) and the hydrazides **2**(**a-c**) of the designed target Schiff bases were obtained commercially, however, the other building blocks were synthesized according to the reported literature [18,19,20,21]. All other chemicals used were of commercially available reagent grade and were used without further purification. Microwave irradiations were carried out using an Explorer-48 microwave reactor (CEM, Matthews, North Carolina, USA). Infrared (IR) Spectra were recorded as KBr disks using the Perkin Elmer FT–IR Spectrum BX apparatus located at the Research Center, College of Pharmacy, King Saud University (Riyadh, Saudi Arabia). Melting points were determined on a Gallenkamp melting point apparatus, and are uncorrected. NMR Spectra were scanned in DMSO-d_6_ on a Bruker NMR spectrophotometer operating at 500 MHz for ^1^H and 125.76 MHz for ^13^C at the Research Center, College of Pharmacy, King Saud University (Riyadh, Saudi Arabia). Chemical shifts are expressed in δ-values (ppm) relative to TMS as an internal standard. Coupling constants (*J*) are expressed in Hz. D_2_O was added to confirm the exchangeable protons. Mass spectra were measured on a Varian MAT CH-5 spectrometer (70 eV) or in Agilent Triple Quadrupole 6410 QQQ LC/MS with ESI (Electrospray ionization) source. Mtb DNA gyrase inhibitory activity assays were carried out at Department of Antimicrobial Drug Discovery, Achillion Pharmaceuticals (New Haven, CT, USA).

### 4.2. General Procedure for the Synthesis of Schiff Bases **3-26**:

**Method 1.** To a mixture of isatin **1a-k** (1 mmol) and hydrazide **2a-k** (1 mmol) in ethanol (25 mL) a few drops of glacial acetic acid were added. The reaction mixture was refluxed for 4-6 h, and then cooled to room temperature. The precipitate was filtered and dried. The crude product was recrystallized from the appropriate solvent to obtain hydrazone compounds **3-26**.

**Method 2.** A solution of isatin **1a-k** (1 mmol) and hydrazide **2a-k** (1 mmol) in ethanol (15 mL) was prepared. Few drops of glacial acetic acid were added and whole reaction mixture was irradiated under microwave irradiation at 110 °C for 5 minutes. The reaction mixture was cooled. The solid separated on cooling was filtered, washed with cold ethanol, dried and recrystallised from the appropriate solvent to obtain hydrazones **3-26**.

*(Z)-N'-(2-Oxoindolin-3-ylidene)isonicotinohydrazide* (**3**). Yield: 56% (Method 1); Mp: 290–293 °C (EtOH/DMF); IR *ν*: 3166, 3057 (2NH), 1702, 1683 (2C=O), 1617 (C=N) cm^−1^; EI MS *m/z (%)*: 301.2 (M^+^ +1, 37), 299.3 (M^+^, 1), 44.9 (100); ^1^H-NMR *δ*: 6.93 (1H, d, *J* = 8.7, C_6_H), 7.45 (1H, d, *J *= 2.3, C_4_H), 7.79–7.83 (2H, m, C_3´_H, C_5´_H), 8.13 (1H, br. s, C_7_H), 8.82–8.83 (2H, m = 4.6, C_2´_H, C_6´_H), 10.97 (1H, s, NH), 11.88 (1H, br. s, NH); ^13^C-NMR *δ*: 112.03 (C_3a_), 116.44 (C_7_), 120.99 (C_3´_, C_5´_), 122.40 (C_4_), 125.65 (C_5_), 126.52 (C_3_, C_6_), 132.22 (C_4´_), 140.27 (C_7a_), 142.88 (C_2´_, C_6´_), 150.15 (C=O), 164.29 (C_2_).

*(Z)-N'-(5-Chloro-2-oxoindolin-3-ylidene)isonicotinohydrazide* (**4**). Yield: 74% (Method 2); Mp: 345–348 °C (EtOH/DMF); IR *ν*: 3166, 3057 (2NH), 1702, 1683 (2C=O), 1617 (C=N) cm^−1^; EI MS *m/z *(%): 301.2 (M^+^ +1, 37), 299.3 (M^+^, 1), 44.9 (100); ^1^H-NMR *δ*: 6.93 (1H, d, *J* = 8.7, C_6_H), 7.45 (1H, d, *J* = 2.3, C_4_H), 7.79–7.83 (2H, m, C_3´_H, C_5´_H), 8.13 (1H, br. s, C_7_H), 8.82–8.83 (2H, m = 4.6, C_2´_H, C_6´_H), 10.97 (1H, s, NH), 11.88 (1H, br. s, NH); ^13^C-NMR *δ*: 112.03 (C_3a_), 116.44 (C_7_), 120.99 (C_3´_, C_5´_), 122.40 (C_4_), 125.65 (C_5_), 126.52 (C_3_, C_6_), 132.22 (C_4´_), 140.27 (C_7a_), 142.88 (C_2´_, C_6´_), 150.15 (C=O), 164.29 (C_2_).

*(Z)-N'-(5-Fluoro-2-oxoindolin-3-ylidene)isonicotinohydrazide* (**5**). Yield: 59% (Method 2); Mp: 346–348 °C (EtOH/DMF); IR *ν*: 3226, 3057 (2NH), 1709, 1677 (2C=O), 1613(C=N) cm^−1^; EI MS *m/z *(%): 284.8 (M^+^ +202), 283.7 (M^+^, 20), 44.9 (100); ^1^H-NMR *δ*: 6.92 (1H, br. s, C_6_H), 7.29 (1H, br. s, C_4_H), 7.85–7.94 (3H, m, C_3´_H, C_5´_H, C_7_H), 8.81–8.83 (2H, br. s, C_2´_H, C_6´_H), 10.87 (1H, s, NH), 11.94 (1H, s, NH).

*(Z)-N'-(5-Methyl-2-oxoindolin-3-ylidene)isonicotinohydrazide* (**6**). Yield: 69% (Method 2); Mp: 319–322 °C (EtOH/DMF); IR *ν*: 3260 (NH), 1706, 1694 (2C=O), 1626 (C=N) cm^−1^; EI MS *m/z *(%): 281.5 (M^+^ +1, 17), 279.5 (M^+^, 15), 47.8 (100); ^1^H-NMR *δ*: 2.31 (3H, s, CH_3_), 6.86 (1H, d, *J* = 8.3, C_7_H), 7.22 (1H, d, *J* = 7.8, C_6_H), 7.43 (1H, br. s, C_4_H), 7.78 (2H, d, *J* = 4.6, C_3´_H, C_5´_H), 8.87 (2H, d, *J* = 4.6, C_2´_H, C_6´_H), 10.72 (1H, s, NH), 11.30 (1H, s, NH); ^13^C-NMR *δ*: 20.48 (CH_3_), 111.10 (C_3a_), 119.56 (C_7_), 120.89 (C_3´_, C_5´_), 121.27 (C_4_), 121.51 (C_3_, C_6_), 131.99 (C5), 132.63 (C_4´_), 139.24 (C_7a_), 140.48 (C_2´_, C_6´_), 150.92 (C=O), 162.98 (C_2_).

*(Z)-N'-(2-Oxo-5-(trifluoromethoxy)indolin-3-ylidene)isonicotinohydrazide* (**7**). Yield: 33% (Method 2); Mp: 262–265 °C (EtOH/DMF); IR *ν*: 3236 (NH), 1712, 1698 (2C=O), 1628 (C=N) cm^−1^; EI MS *m/z *(%): 351.6 (M^+^ +1, 0.6), 350.3 (M^+^, 4), 40.1 (100); ^1^H-NMR *δ*: 7.05–7.07 (1H, m, C_7_H), 7.41–7.54 (2H, m, C_4_H, C_6_H), 7.71–7.79 (2H, m, C_3´_H, C_5´_H), 8.79–8.87 (2H, m, C_2´_H, C_6´_H), 11.59 (1H, br. s, NH); ^13^C-NMR *δ*: 112.49 (C_3a_), 114.37 (C_7_), 120.90 (C_3´_, C_5´_), 121.04(CF_3_), 121.17 (C_4_), 125.14 (C5), 138.28 (C_6_), 138.89 (C_3_), 138.97 (C_4´_), 141.67 (C_2´_, C_6´_), 143.71 (C_7a_), 150.93 (C=O), 163.04 (C_2_).

*(Z)-N'-(5-Nitro-2-oxoindolin-3-ylidene)isonicotinohydrazide* (**8**). Yield: 70% (Method 2); Mp: 350–353 °C (EtOH/DMF); IR *ν*: 3328, 3184 (2NH), 1739, 1709 (2C=O), 1606 (C=N) cm^−1^; EI MS *m/z *(%): 313.0 (M^+^ +2, 2.5), 311.0 (M^+^, 17), 78.0 (100); ^1^H-NMR *δ*: 7.67 (1H, br. s, C_7_H), 7.71–7.79 (2H, m, C_3´_H, C_5´_H), 8.79–8.87 (2H, m, C_2´_H, C_6´_H), 8.31 (2H, br. s, C_4_H, C_6_H), 11.48 (1H, br. s, NH), 12.04 (1H, br. s, NH).

*(Z)-N'-(2-Oxo-1-phenylindolin-3-ylidene)isonicotinohydrazide* (**9**). Yield: 90% (Method 2); Mp: 201–205 °C; IR *ν*: 3296 (NH), 1708, 1688 (2C=O), 1613 (C=N) cm^−1^; EI MS *m/z *(%): 342.1 (M^+^, 25), 40.1 (100); ^1^H-NMR *δ*: 6.88 (1H, d, *J* = 8, C_d_ H), 7.25 (1H, t, *J* = 8, C_e_ H), 7.46 (1H, t, *J* = 7.5, C_f_ H), 7.53–7.74 (8H, m, C_g_ H, C_3 + 3*_ H and H of ph), 8.86 (2H, d, J = 3, C_2 + 2*_ H), 13.91 (1H, s, NHN=); ^13^C-NMR *δ*: 110.31 (C_c*_), 119.12 (C_e_), 121.10 (C_4-ph_), 121.25 (C_2-ph+6-ph_), 123.87 (C_3 + 3*_), 126.64 (C_d+g+5-ph_), 128.74 (C_4-ph_), 129.74 (C_f+c_), 132.12 (C_1-ph_), 132.65 (C_g*_), 139.01 (C_4_), 143.89 (C_2 + 2*_), 150.92 (C_b_), 160.55 (C_5_).

*(Z)-N'-(1-Benzyl-2-oxoindolin-3-ylidene)isonicotinohydrazide* (**10**). Yield: 20% (Method 1); Mp: 162–165 °C (EtOH); IR *ν*: 3224 (NH), 1703, 1676 (2C=O), 1614(C=N) cm^−1^; EI MS *m/z *(%): 356.1 (M^+^, 7.5), 40.1 (100); ^1^H- NMR *δ*: 5.03 (2H, s, CH_2_), 7.31–7.41 (9H, m, H_ar._), 7.82 (2H, br. s, C_3´_H, C_5´_H), 8.87 (2H, m, C_2´_H, C_6´_H); ^13^C-NMR *δ*: 42.50 (CH_2_), 110.64 (C_3a_), 119.10 (C_7_), 121.06 (C_3´_, C_5´_), 123.54, 127.46, 127.68, 128.72, 132.01, 135.55, 142.93 (C_ar._, C_2´_, C_4´_, C_6´_), 150.89 (C=O), 161.07 (C_2_).

*(Z)-N'-(1-Benzyl-5-chloro-2-oxoindolin-3-ylidene)isonicotinohydrazide* (**11**). Yield: 26% (Method 1); Mp: 167–171 °C (EtOH); IR *ν*: 3447 (NH), 1701, 1689 (2C=O), 1616 (C=N) cm^−1^; EI MS *m/z *(%): 390 (M^+^, 3.2), 47.3 (100); ^1^H- NMR *δ*: 5.03 (2H, s, CH_2_), 7.31–7.39 (8H, m, H_ar._), 7.81 (2H, d, *J* = 7.8, C_3´_H, C_5´_H), 8.86 (2H, d, *J* = 7.8, C_2´_H, C_6´_H); ^13^C-NMR *δ*: 43.01 (CH_2_), 112.15 (C_3a_), 120.55, 120.96, 121.28, 127.43, 127.72, 128.72, 131.24, 135.31, 138.99, 141.53 (C_ar._), 150.89 (C=O), 161.50 (C_2_).

*(Z)-N'-(1-Benzyl-5-fluoro-2-oxoindolin-3-ylidene)isonicotinohydrazide* (**12**). Yield: 17% (Method 1); Mp: 188–192 °C (EtOH); IR *ν*: 3446 (NH), 1709, 1683 (2C=O), 1625 (C=N) cm^−1^; EI MS *m/z *(%): 374.8 (M^+^, 3.2), 62.2 (100); ^1^H-NMR *δ*: 5.03 (2H, s, CH_2_), 6.91 (1H, dd, *J *= 8.93, 4.36, C_6_H), 7.11–7.64 (6H, m, C_4_H, H_ar._), 7.71–7.79 (2H, m, C_3´_H, C_5´_H), 7.99 (1H, d, *J *= 7.36, C_7_H), 8.79–8.87 (2H, m, C_2´_H, C_6´_H),11.88 (1H, s, NH).

*(Z)-N'-(1-Benzyl-5-methyl-2-oxoindolin-3-ylidene)isonicotinohydrazide* (**13**). Yield: 27% (Method 1); Mp: 159–162 °C (EtOH); IR *ν*: 3448 (NH), 1703, 1682 (2C=O), 1623 (C=N) cm^−1^; EI MS *m/z *(%): 370.0 (M^+^, 7.5), 44.9 (100); ^1^H-NMR *δ*: 2.31 (3H, s, CH_3_), 4.99 (2H, s, CH_2_), 6.98 (1H, d, *J* = 8.24, C_7_H), 7.21–7.40 (6H, m, H_ar._), 7.50 (1H, br. s, C_4_H), 7.81–7.85 (2H, m, C_3´_H, C_5´_H), 8.87–8.89 (2H, m, C_2´_H, C_6´_H).

(*Z*)-*N*'-(2-Oxoindolin-3-ylidene)nicotinohydrazide (**14**). Yield: 72% (Method 1); Mp: 254–256 °C (EtOH/DMF); IR *ν*: 3226 (NH), 1719, 1695 (2C=O), 1620 (C=N) cm^−1^; EI MS *m/z *(%): 267.2 (M^+^ +1, 7), 265.9 (M^+^, 15), 64.0 (100); ^1^H-NMR (DMSO-*d_6_*) *δ*: 6.97 (1H, d, *J* = 7.8, C_4_H), 7.12 (1H, t, *J* = 7.8, C_5_H), 7.41 (1H, t, *J* = 7.8, C_6_H), 7.64–7.67 (2H, m, C_7_H, C_5´_H), 8.26 (1H, d, *J *= 5.1 C_4´_H), 8.85 (1H, d, *J* = 6.1, C_6´_H), 9.05 (1H, s, C_2´_), 11.39 (1H, s, NH); ^13^C-NMR *δ*: 111.29 (C_3a_), 119.64 (C_7_), 121.08, 122.81, 124.05, 128.06, 132.03, 142.60, 148.5 (C_ar._), 153.12 (C=O), 162.94 (C_2_).

*(Z)-N'-(5-Fluoro-2-oxoindolin-3-ylidene)furan-2-carbhydrazide* (**15**). Yield: 57% (Method 2); Mp: 310–312 °C (EtOH/DMF); IR *ν*: 3447, 3160 (2NH), 1715, 1667(2C=O), 1611 (C=N) cm^−1^; EI MS *m/z *(%): 272.8 (M^+^, 6), 40.1 (100); ^1^H-NMR *δ*: 6.78–6.79 (1H, m, H_ar._), 6.89–6.90 (1H, m, H_ar._), 7.25–7.29 (1H, m, H_ar._), 7.70 (1H, br. s, H_ar._), 7.94 (1H, br. s, H_ar._), 8.07 (1H, s, H_ar._), 10.87 (1H, s, NH), 11.67 (1H, s, NH); ^13^C-NMR (DMSO-*d_6_*) *δ*: 111.49 (C_4´_), 112.47 (C_3´_), 113.55 (C_4_), 115.76 (C_3a_), 118.97 (C_6_), 119.68 (C_7_), 135.01, 140.28, 145.26, 147.26 (C_ar._), 156.15 (C=O), 164.78 (C_2_).

*(Z)-5-Nitro-N'-(2-Oxoindolin-3-ylidene)furan-2-carbohydrazide* (**16**). Yield: 82% (Method 2); Mp: 309–11 °C (EtOH/DMF); ESI MS *m/z*: 299.1 [M − 1]^−^; ^1^H-NMR *δ*: 6.79 (1H, s, H_ar._), 6.97–6.99 (1H, m, H_ar._), 7.13–7.15 (1H, m, H_ar._), 7.41–7.43 (1H, m, H_ar._), 7.64–7.67 (2H, m, H_ar._), 7.88 (1H, br. s, H_ar._), 11.38 (1H, s, NH).

*(Z)-N'-(5-Chloro-2-oxoindolin-3-ylidene)benzofuran-2-carbohydrazide* (**17**). Yield: 85% (Method 2); Mp: 335–338 °C (EtOH/DMF); ESI MS *m/z*: 340.1 [M + 1]^+^; ^1^H-NMR *δ*: 6.94 (1H, d, *J* = 8.08, H_ar._), 7.38–7.56 (3H, m, H_ar._), 7.75 (1H, d, *J* = 8.76, H_ar._), 7.89 (1H, d, *J* = 8.04, H_ar._), 8.09 (1H, s, H_ar._), 8.19 (1H, s, H_ar._), 11.03 (1H, s, NH), 12.03 (1H, s, NH); ^13^C-NMR *δ*: 112.54 (C_3´_), 112.61 (C_7´_), 117.09 (C_3a_), 121.28, 124.01, 124.63, 126.29, 126.77, 127.51, 128.53, 128.82, 132.01, 132.82, 143.39, 155.08 (C_ar._), 163.25 (C=O), 165.03 (C_2_).

*(Z)-N'-(5-Methyl-2-oxoindolin-3-ylidene)benzofuran-2-carbohydrazide* (**18**). Yield: 89% (Method 2); Mp: 331–36 °C (EtOH/DMF); ESI MS *m/z*: 320.2 [M + 1]^+^; ^1^H-NMR *δ*: 2.32 (CH_3_), 6.83 (1H, d, *J* = 8.08, H_ar._), 7.21–7.26 (1H, m, H_ar._), 7.38–7.40 (1H, m, H_ar._), 7.47–7.59 (2H, m, H_ar._), 7.76 (1H, t, *J* = 7.70, H_ar._), 7.85–7.90 (2H, m, H_ar._), 10.78 (1H, s, NH), 11.77 (1H, s, NH); ^13^C-NMR *δ*: 21.07 (CH_3_), 111.09 (C_3´_), 111.62 (C_7´_), 112.59 (C_3a_), 120.27, 122.11, 123.91, 124.60, 127.55, 128.69, 131.32, 132.47, 133.09, 133.88, 141.07, 155.11 (C_ar._), 163.51 (C=O), 165.30 (C_2_).

*(Z)-N'-(5-Nitro-2-oxoindolin-3-ylidene)benzofuran-2-carbohydrazide* (**19**). Yield: 79% (Method 2); Mp: 367–69 °C (EtOH/DMF); ESI MS *m/z*: 351.1 [M + 1]^+^; ^1^H-NMR *δ*: 7.10 (1H, d, *J* = 8.80, H_ar._), 7.41 (1H, t, *J* = 7.70, H_ar._), 7.58 (1H, t, *J* = 7.70, H_ar._), 7.75 (1H, d, *J* = 8.08, H_ar._), 7.85–7.92 (2H, m, H_ar._), 8.07 (1H, s, H_ar._), 8.29–8.63 (1H, m, H_ar._), 9.03(1H, s, NH), 11.59 (1H, s, NH).; ^13^C-NMR *δ*: 111.39 (C_3´_), 112.48 (C_7´_), 112.62 (C_3a_), 121.01, 123.96, 124.04, 124.67, 127.50, 128.34, 128.65, 128.93, 142.51, 143.44, 148.35, 150.05, 155.07 (C_ar._), 163.78 (C=O), 165.58 (C_2_).

*(Z)-N'-(2-Oxo-1-phenylindolin-3-ylidene)benzofuran-2-carbohydrazide* (**20**). Yield: 88% (Method 2); Mp: 235–38 °C (EtOH/DMF); ESI MS *m/z*: 382.3 [M + 1]^+^; ^13^C-NMR (DMSO-*d_6_*) *δ*: 110.81 (C_3´_), 112.63 (C_7´_), 112.69 (C_3a_), 115.95, 119.83, 121.82, 123.88, 124.37, 124.86, 127.39, 127.52, 128.82, 129.34, 130.28, 130.35, 132.59, 133.36, 144.52, 152.60 (C_ar._), 155.15 (C_2_), 161.15 (C=O).

*(Z)-N'-(5-Fluoro-2-oxoindolin-3-ylidene)-5-nitrobenzofuran-2-carbhydrazide* (**21**). Yield: 86% (Method 2); Mp: 357–59 °C (EtOH/DMF); ESI MS *m/z*: 367.1 [M − 1]^−^; ^1^H-NMR *δ*: 6.92 (1H, dd, *J* = 4.40, 8.76, H_ar._), 6.99 (1H, dd, *J* = 3.68, 8.08, H_ar._), 7.25–7.29 (1H, m, H_ar._), 7.37–7.43 (1H, m, H_ar._), 7.49–7.59 (1H, m, H_ar._), 7.76 (1H, d, *J* = 8.80, H_ar._), 8.0–8.12 (1H, m, H_ar._), 8.25–8.42 (1H, m, H_ar._), 10.88 (1H, s, NH), 11.97 (1H, s, NH).

*(Z)-N'-(2-oxo-1-phenylindolin-3-ylidene)adamantane-1-carbohydrazide* (**22**). Yield: 56% (Method 2); Mp: 201–203 °C (EtOH/DMF); ESI MS *m/z*: 400.2 [M + 1]^+^, 422.2 (M + 23 (Na)]^+^; ^1^H-NMR *δ*: 1.71–2.03 (15H, m, adamantine 6CH_2_ and 3CH) 6.84 (1H, br. s, H_ar._), 7.23–7.68 (8H, m, H_ar._).

*(Z)-N'-(1-Benzyl-2-oxoindolin-3-ylidene)-2-(2,3-dimethylphenylamino)benzohydrazide* (**23**). Yield: 37% (Method 1); Mp: 286–288 °C (EtOH); ESI MS *m/z*: 475.2 [M + 1]^+^; ^1^H-NMR *δ*: 2.14 (3H, s, CH_3_), 2.29 (3H, s, CH_3_), 5.03 (2H, s, CH_2_), 6.82–6.89 (2H, m, H_ar._), 7.01–7.19 (5H, m, H_ar._), 7.29–7.39 (7H, m, H_ar._), 7.66–7.70 (2H, m, H_ar._), 9.23 (1H, s, NH).

*(Z)-2-(2,6-Dichloro-3-methylphenylamino)-N'-(5-methyl-2-oxoindolin-3-ylidene)-benzohydrazide* (**24**).

Yield: 33% (Method 1); Mp: 283–287 °C (EtOH/DMF); ESI MS *m/z*: 453.1 [M]^+^; ^1^H-NMR *δ*: 2.32 (3H, s, CH_3_), 2.39 (3H, s, CH_3_), 6.33 (1H, d, *J* = 8.24, H_ar._), 6.87 (1H, d, *J* = 8.24, H_ar._), 6.94 (1H, t, *J *= 7.78, H_ar._), 7.21 (1H, d, *J* = 8.24, H_ar._), 7.34–7.41 (2H, m, H_ar._), 7.44 (1H, s, H_ar._), 7.52 (1H, d, *J* = 8.24, H_ar._), 7.68 (1H, d, *J* = 8.24, H_ar._), 9.46 (1H, s, NH), 11.26 (1H, s, NH); ^13^C-NMR *δ*: 20.15 (CH_3_), 20.57 (CH_3_), 111.05, 114.44, 118.39, 119.90, 121.28, 128.17, 129.11, 131.89, 132.25, 133.79, 136.56, 140.24, 146.39 (C_ar._), 163.12 (C=O), 166.01 (C_2_).

*(Z)-N'-(2-Oxo-5-(trifluoromethoxy)indolin-3-ylidene)-2-(3-(trifluoromethyl)phenylamino)-benzo-hydrazide* (**25**). Yield: 45% (Method 1); Mp: 226 °C (EtOH/DMF); ESI MS *m/z*: 509.1 [M + 1]^+^, 531.1 [M + 23 (Na)]^+^; ^1^H-NMR *δ*: 6.99 (1H, d, *J* = 7.32, H_ar._), 7.17–7.76 (10H, m, H_ar._), 9.02 (1H, s, NH), 11.44 (1H, s, NH).

*(Z)-1-Ethyl-N'-(5-fluoro-2-oxoindolin-3-ylidene)-7-methyl-4-oxo-1,4-dihydro-1,8-naphthyridine-3-carbohydrazide* (**26**). Yield: 55% (Method 2); Mp: >360 °C (EtOH/DMF); IR *ν*: 3070 (2NH), 1723–1685 (3C=O), 1608 (2C=N) cm^−1^; EI MS *m/z *(%): 392.9 (M^+^, 5), 40.1 (100); ^1^H-NMR *δ*: 1.58 (3H, t, *J* = 6.5, N_1_'CH_2_CH_3_), 2.73 (3H, s, C_7_'CH_3_), 4.63 (2H, q, *J* = 7.5, N_1_'CH_2_CH_3_), 6.98 (1H, dd, *J* = 8.93, 4.36, C_6_H), 7.34-7.60 (1H, m, C_4_H), 8.59 (1H, d, *J* = 7.5, C_6_'H), 8.72 (1H, d, *J* = 7.36, C_7_H), 8.98 (1H, d, *J* = 8.5, C_5_'H), 9.32 (1H, s, C_2_'H), 10.86 (1H, s, NH).

### 4.3. Mycobacterium Tuberculosis (Mtb) Gyrase Supercoiling Assay

Relaxed pBR322 DNA (0.1 µg) was incubated with 1 unit of Mtb DNA gyrase for 60 minutes at 37 °C in the following buffer: 2 mM ATP, 4 mM DTT, 25 mM KCl, 6 mM magnesium acetate, 50 µg/mL of molecular grade BSA, 0.1 mg/mL E. coli tRNA, 2mM spermidine, 100 mM potassium glutamate and 40 mM Tris-HCL pH 7.5 (20 µL). Reactions are stopped with 0.5% SDS, 6 mM EDTA, 5.35% glycerol, and 0.013% bromophenol blue (10 µL). Reactions are then loaded onto a 1% agarose/ TBE gel to separate supercoiled and relaxed DNA forms and run overnight at 22 volts. Bands are visualized by staining with 0.5 µg/mL EtBr in 1 × TBE for 40 minutes then washing with dH_2_O for 120 minutes. Quantification is done on the AlphaImager 2200 system. DNA gyrase subunits, GyrA and GyrB, were isolated from Mtb strain H37RV genomic DNA, expressed and purified in pET overexpression constructs in E coli. One unit of DNA supercoiling activity is defined as the amount that converts ~50% relaxed DNA, in the standard assay, to supercoiled DNA in 60 minutes at 37 °C.
